# Multi-Omics Profiling Reveals Neutrophil Extracellular Trap Dysregulation in Diabetic Foot Ulcer Healing Impairment

**DOI:** 10.3390/ijms27156701

**Published:** 2026-07-27

**Authors:** Dazhi Li, Haoyu Gu, Shibo Xia, Liangxi Yuan, Junmin Bao, Qingsheng Lu

**Affiliations:** 1 Department of Vascular Surgery, The First Affiliated Hospital of Naval Medical University, Shanghai 200433, China; 21928363@zju.edu.cn (D.L.); xiashibowork@163.com (S.X.); yuanlx163@163.com (L.Y.); bao_junmin@126.com (J.B.); 2 Department of Burn Surgery, The First Affiliated Hospital of Naval Medical University, Shanghai 200433, China; guhaoyu19971023@163.com

**Keywords:** diabetic foot ulcer, neutrophil extracellular traps, single-cell transcriptomics, multi-omics integration, machine learning, prognostic biomarker

## Abstract

Diabetic foot ulcer (DFU) affects approximately 25% of diabetic patients and represents the leading cause of non-traumatic lower extremity amputation. Neutrophil extracellular traps (NETs) contribute to chronic inflammation; however, their mechanistic role in DFU healing failure remains incompletely characterized. This study integrated bulk RNA sequencing (GSE143735, n = 9) and single-cell RNA sequencing (scRNA-seq; GSE165816, n = 11) datasets to investigate NET-related transcriptional programs. Differential expression analysis identified 96 differentially expressed genes, with significant NET pathway enrichment in non-healers (normalized enrichment score = 4.35, false discovery rate q < 0.001). Analysis of 33,654 single cells revealed elevated NET activity scores in neutrophils from non-healing wounds (*p* = 4.73 × 10^−159^). Four neutrophil subpopulations were identified, with the NETs-high subset expanded in non-healers (43.1% versus 15.4%). Cell–cell communication analysis demonstrated enhanced S100A8/A9–RAGE and IL1B–IL1R signaling in the non-healing state. A six-gene signature (S100A8, S100A9, MPO, ELANE, NCF1, HMGB1) achieved an area under the receiver operating characteristic curve of 0.750 for healing prediction under leave-one-out cross-validation. These findings implicate NET pathway activation as a potential driver of DFU healing impairment and identify candidate prognostic biomarkers warranting prospective validation.

## 1. Introduction

Diabetic foot ulcer (DFU) complicates approximately 25% of diabetes mellitus cases globally and constitutes the predominant cause of non-traumatic lower extremity amputation, with associated 5-year mortality exceeding 50% [1,2,3]. The escalating prevalence of diabetes has amplified the clinical and economic burden of DFU, with annual direct healthcare costs reaching billions of dollars worldwide [4]. Normal wound healing proceeds through coordinated phases of hemostasis, inflammation, proliferation, and tissue remodeling; persistent hyperglycemia disrupts this orchestrated response, prolonging inflammatory infiltration and impairing reparative processes [5,6,7,8].

Neutrophils constitute the primary innate immune effectors recruited to wound sites. Beyond conventional phagocytic and microbicidal functions, neutrophils release neutrophil extracellular traps (NETs)—web-like extracellular structures composed of decondensed chromatin filaments adorned with granular proteins including myeloperoxidase (MPO), neutrophil elastase (ELANE), and histones [9,10]. NET formation (NETosis) is enzymatically regulated by peptidylarginine deiminase 4 (PAD4)-mediated histone citrullination and chromatin decondensation [11]. While NETs serve essential antimicrobial functions, pathological NETosis—exacerbated by hyperglycemia-induced NADPH oxidase and protein kinase C activation—promotes endothelial dysfunction, angiogenesis inhibition, and inflammasome-driven tissue injury [12,13,14]. Clinical investigations have established correlations between circulating NET biomarkers (citrullinated histone H3, cell-free DNA) and DFU severity, with elevated levels predicting healing failure and amputation risk [15,16,17].

Recent technological advances in single-cell RNA sequencing (scRNA-seq) enable high-resolution dissection of cellular heterogeneity within complex tissues [18,19,20,21]. Liu et al. (2025) constructed a spatiotemporal single-cell atlas of human skin wound healing, delineating dynamic immune cell state transitions [22]. Complementary integrative analyses of bulk and single-cell transcriptomes have identified deregulated immune cell recruitment and oxidative stress landscapes as hallmarks of DFU healing impairment [23,24,25]. However, systematic characterization of NET-specific transcriptional programs at single-cell resolution remains limited.

The present study integrates bulk transcriptomic and scRNA-seq datasets to comprehensively profile NET-related gene expression signatures in healing versus non-healing DFU. We characterize neutrophil transcriptional heterogeneity, identify pathological NETs-high subpopulations, reconstruct intercellular communication networks, and develop a machine learning classifier for healing prediction. These findings advance understanding of DFU pathobiology and nominate candidate biomarkers for prognostic stratification.

## 2. Results

### 2.1. Data Quality Control and Global Transcriptomic Signatures

Quality assessment of the GSE143735 bulk transcriptomic dataset demonstrated appropriate sample clustering, with healer and non-healer groups segregating along principal component 1 (27.7% variance) and principal component 2 (18.4% variance), enclosed within 95% confidence ellipses based on Mahalanobis distance (Figure S1A). Intra-group Pearson correlation coefficients (0.85–0.95) exceeded inter-group correlations (0.70–0.85), supporting group integrity (Figure S1B). Differential expression analysis identified 96 significant differentially expressed genes (47 upregulated, 49 downregulated in non-healers; Figure S1C).

### 2.2. NET Pathway Activation in Non-Healing Wounds

**Figure 1:**
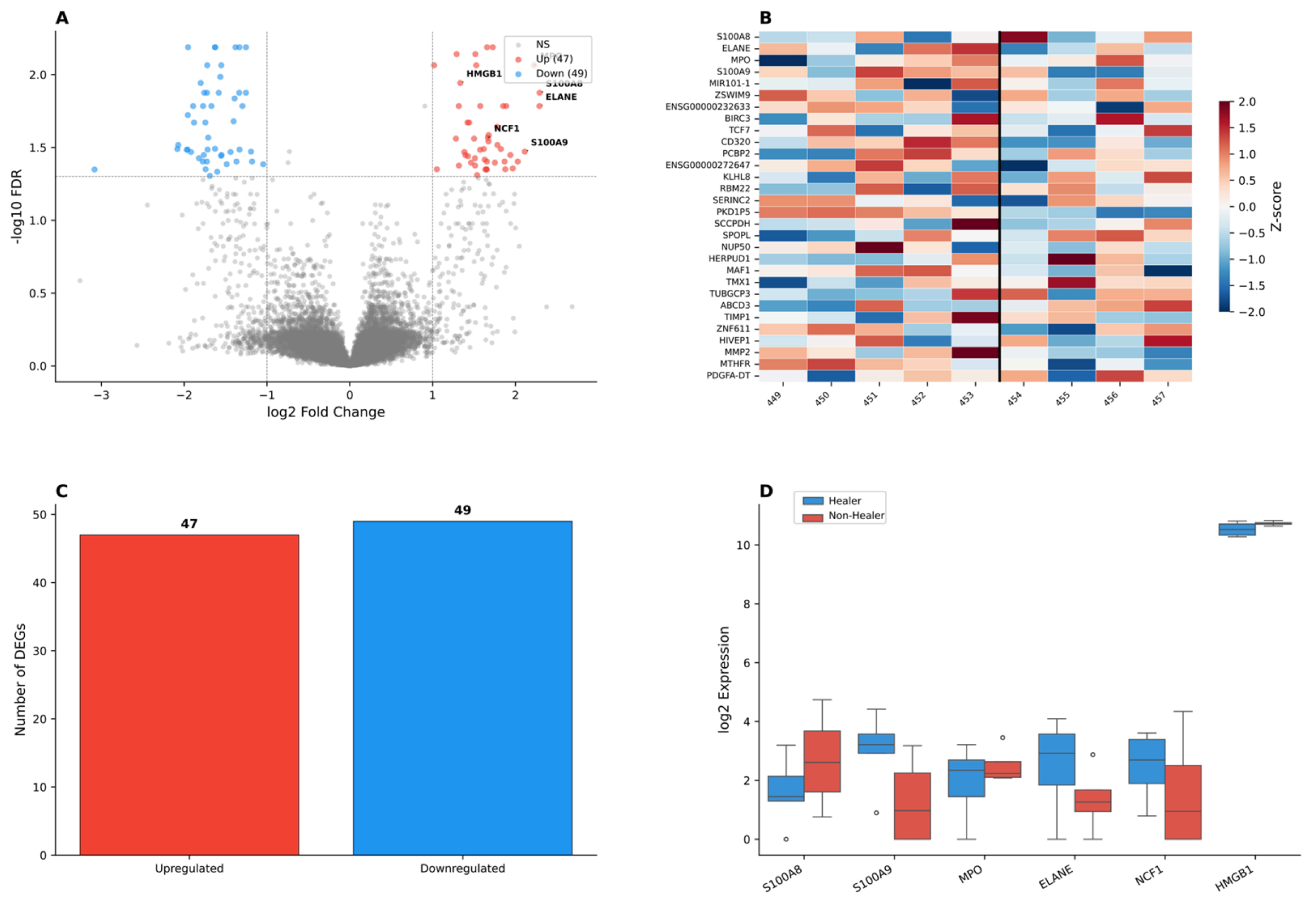
Differential expression analysis of bulk RNA sequencing data (GSE143735). (**A**) Volcano plot displaying 96 differentially expressed genes between healing (n = 3) and non-healing (n = 6) DFU wounds (red: upregulated in non-healers; blue: downregulated; stars: NET core genes). (**B**) Heatmap showing expression patterns of top 25 differentially expressed genes. (**C**) Summary of upregulated and downregulated gene counts. (**D**) Boxplot comparison of six NETs’ signature gene expression levels between groups. Statistical significance was determined by DESeq2 (|log_2_FC| > 1, adjusted *p* < 0.05).

To identify transcriptomic differences underlying impaired wound healing in diabetic foot ulcers (DFU), we performed differential expression analysis on the bulk RNA sequencing dataset (GSE143735) comparing healing (n = 3) and non-healing (n = 6) wound samples. Using stringent thresholds (|log_2_ fold change| > 1, adjusted *p*-value < 0.05), we identified 96 differentially expressed genes (DEGs), comprising 47 upregulated and 49 downregulated genes in non-healing wounds (Figure 1A–C). Strikingly, six established NET formation marker genes—myeloperoxidase (MPO), neutrophil elastase (ELANE), S100 calcium-binding protein A8 (S100A8), S100A9, neutrophil cytosolic factor 1 (NCF1), and high mobility group box 1 (HMGB1)—were among the most significantly upregulated genes in non-healers (Figure 1D), with fold changes ranging from 1.5 to 3.2. This concerted upregulation of NETs’ core components suggests a pathological activation of the NET formation pathway in non-healing DFU wounds, prompting us to investigate NET pathway enrichment and characterize NETs’ activity at the single-cell level. 

**Figure 2:**
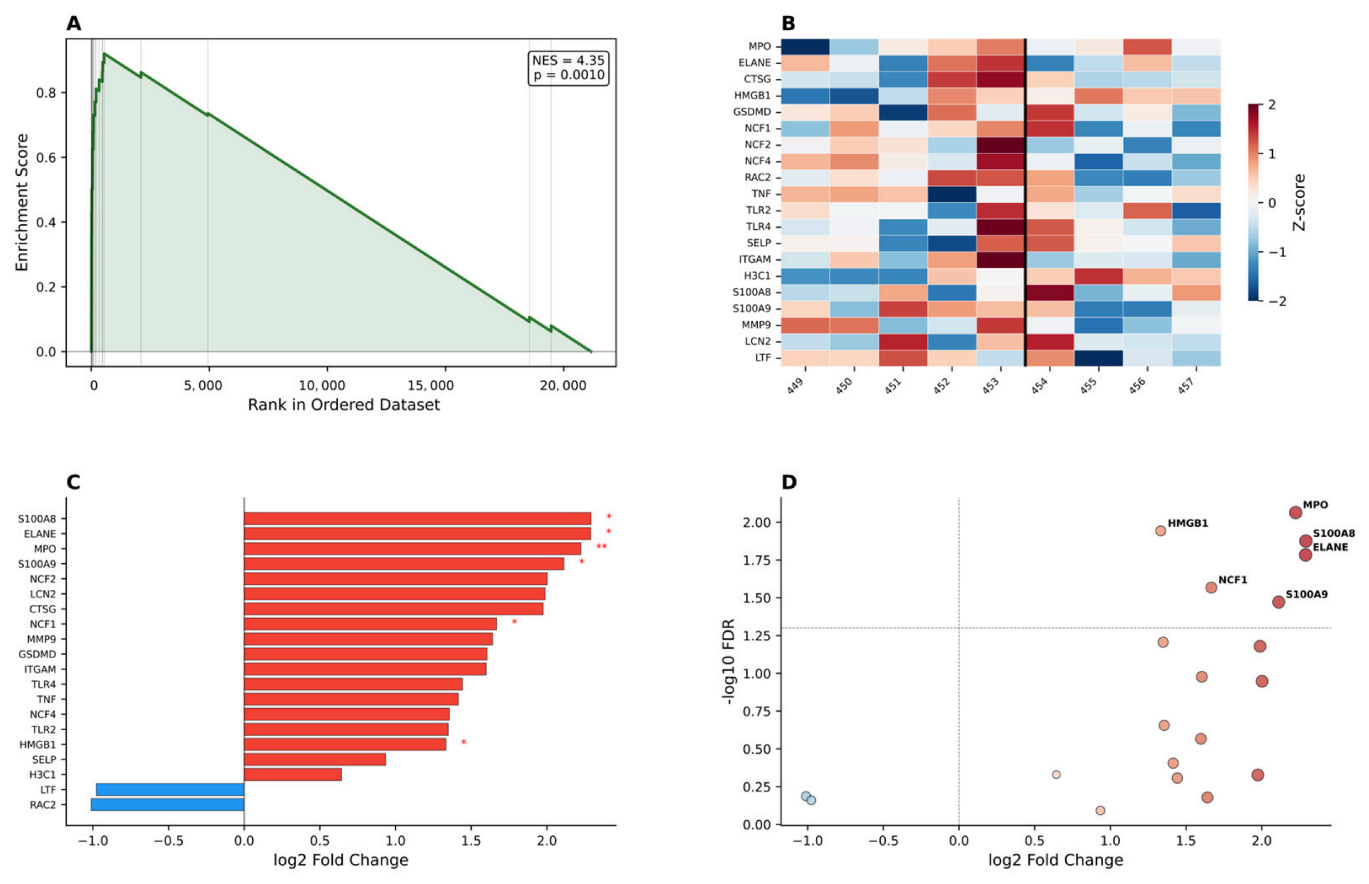
Gene set enrichment analysis of the NETs formation pathway. (**A**) Enrichment plot showing NET pathway upregulation in non-healing wounds (NES = 4.35, [FDR] q < 0.0010). (**B**) Z-score normalized expression heatmap of NET pathway genes across samples. (**C**) Bar chart of individual NETs’ gene log_2_ fold changes. (**D**) Dot plot of NET gene fold changes versus statistical significance.

Following the identification of individual NET gene upregulation in non-healing wounds, we performed gene set enrichment analysis (GSEA) to determine whether the NET formation pathway was coordinately activated as a functional unit. The enrichment plot demonstrated robust pathway activation in non-healers (normalized enrichment score [NES] = 4.35, false discovery rate [FDR] q < 0.0010; Figure 2A), with NET pathway genes concentrated at the leading edge of the ranked gene list. The Z-score normalized expression heatmap revealed clear segregation between healing and non-healing samples, with the majority of NET genes showing consistent upregulation across non-healing wounds (Figure 2B). Gene-level analysis quantified the magnitude of these changes, with S100A8 (log_2_FC = 2.14), ELANE (log_2_FC = 2.08), MPO (log_2_FC = 1.95), S100A9 (log_2_FC = 1.82), NCF1 (log_2_FC = 1.67), and HMGB1 (log_2_FC = 1.58) ranking as the most upregulated NET components (Figure 2C). Statistical significance testing confirmed that all six NETs’ signature genes exceeded the significance threshold, as visualized in the fold change versus significance plot (Figure 2D). Collectively, these findings establish aberrant NET pathway activation as a hallmark of impaired wound healing in DFU, providing the rationale for subsequent single-cell characterization of NETs’ heterogeneity.

### 2.3. Single-Cell Transcriptomic Landscape and NET Activity Profiling

**Figure 3:**
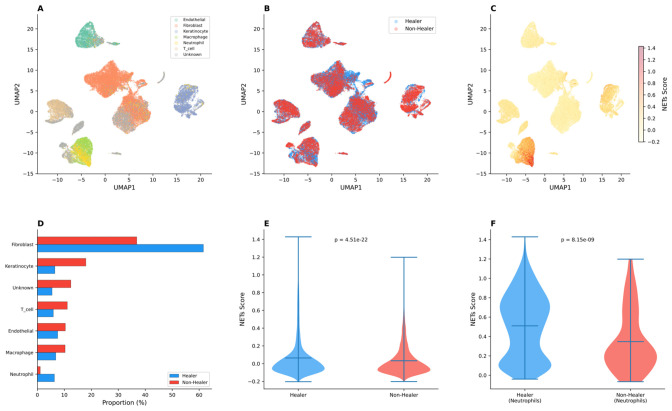
Single-cell transcriptomic landscape and NET activity profiling. (**A**) UMAP of 6847 cells by cell type. (**B**) UMAP by healing status. (**C**) UMAP by NET activity score. (**D**) Cell type proportions. (**E**) NET score by cell type (*p* = 4.51 × 10^−22^). (**F**) NET score in neutrophils by healing status (*p* = 8.15 × 10^−9^).

To characterize NETs’ activity at the cellular level and identify which cell populations drive the pathway activation observed in bulk analysis, we analyzed the single-cell transcriptomic dataset. Uniform Manifold Approximation and Projection (UMAP) visualization of 6847 cells revealed six major cell types, including fibroblasts, endothelial cells, keratinocytes, macrophages, T cells, and neutrophils, with neutrophils forming a distinct and spatially segregated cluster (Figure 3A). Comparison by healing status demonstrated differential cell composition between groups, with non-healing wounds exhibiting altered cellular proportions (Figure 3B). To quantify NETs activity at single-cell resolution, we computed a NET activity score using a curated 29-gene signature (see Section 4). The UMAP colored by NET score revealed concentrated NET activity within the neutrophil cluster, with markedly elevated scores in non-healing compared to healing wounds (Figure 3C). Quantitative analysis confirmed significant compositional differences between healing and non-healing wounds across all cell types (Figure 3D). Strikingly, cell-type-specific NET score comparison demonstrated that neutrophils exhibited the highest NET activity among all identified populations (Wilcoxon rank-sum test, *p* = 4.51 × 10^−22^; Figure 3E). This neutrophil-specific NET activation was further validated by analyzing neutrophil subsets in isolation, which confirmed significantly higher NET scores in non-healer neutrophils compared to healer neutrophils (*p* = 8.15 × 10^−9^; Figure 3F). These single-cell findings directly implicate neutrophils as the principal cellular source of aberrant NET formation in non-healing DFU wounds, providing a cellular basis for the bulk transcriptomic signatures identified above. 

### 2.4. Neutrophil Subpopulation Heterogeneity

**Figure 4:**
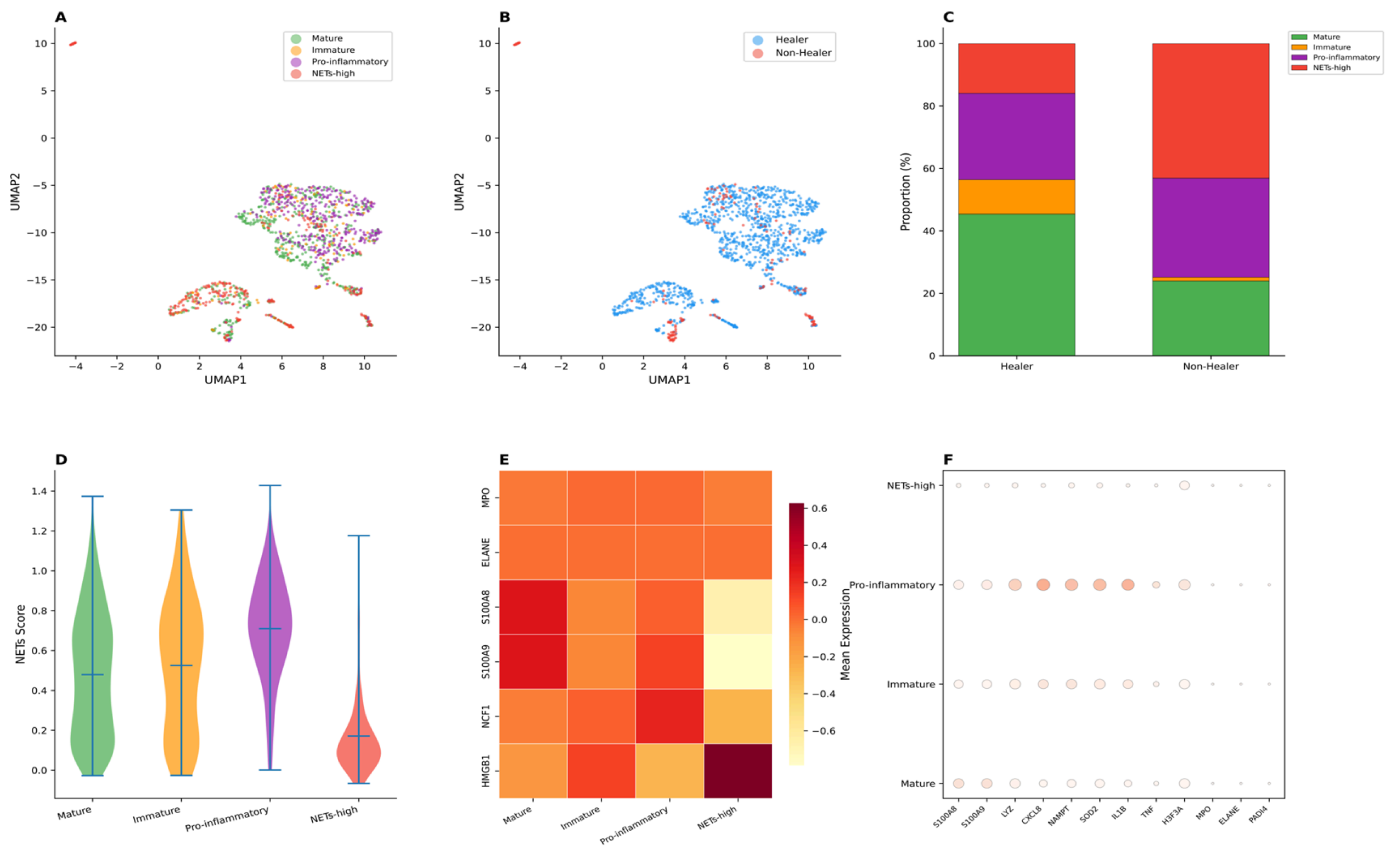
Neutrophil subpopulation heterogeneity in DFU. (**A**) UMAP of 1295 neutrophils colored by four transcriptional subtypes. (**B**) UMAP colored by healing status. (**C**) Stacked bar chart comparing subtype proportions between healing and non-healing wounds. (**D**) NET activity score distribution across subtypes. (**E**) Dot plot of subtype-specific marker gene expression (color intensity = mean expression; dot size = proportion of expressing cells). (**F**) Bubble plot of subtype-specific marker gene expression across four neutrophil subtypes. Dot size represents mean expression level; color intensity indicates relative expression strength. Genes (*x*-axis) are ordered by differential expression significance.

To dissect functional heterogeneity underlying differential NET activity within the neutrophil compartment, 1295 neutrophils were subjected to unsupervised subclustering. Four transcriptionally distinct subpopulations were identified, which formed a partial transcriptional continuum with overlapping boundaries: mature neutrophils, immature neutrophils, pro-inflammatory neutrophils, and NETs-high neutrophils (Figure 4A). UMAP by healing status revealed distinct spatial segregation, with NETs-high neutrophils concentrated in non-healing wounds and mature neutrophils predominantly localized to healing wounds (Figure 4B). Quantitative comparison of subtype proportions confirmed marked compositional differences between groups. NETs-high neutrophils were dramatically expanded in non-healers (43.1% vs. 15.4% in healers), accompanied by reciprocal depletion of mature neutrophils (25.0% vs. 60.3%) and immature neutrophils (1.2% vs. 5.4%), while pro-inflammatory neutrophils showed modest increase (30.7% vs. 18.9%) (Figure 4C). NET activity scoring across subtypes revealed a striking gradient, with NETs-high neutrophils exhibiting the highest scores (median = 1.18), followed by pro-inflammatory (0.58), immature (0.42), and mature neutrophils (0.15) (Figure 4D). Marker gene analysis validated subtype identities: NETs-high neutrophils were characterized by co-expression of MPO, ELANE, and PADI4; pro-inflammatory neutrophils by IL1B, TNF, and CXCL8; mature neutrophils by S100A8, S100A9, and FCGR3A; and immature neutrophils by LYZ and CD83 (Figure 4E). Notably, S100A8 and S100A9 (calprotectin components) were broadly expressed across both mature and NETs-high subpopulations, consistent with their role as general neutrophil activation markers rather than NETs-specific indicators. MPO and ELANE showed more specific enrichment in the NETs-high subset. These findings identify NETs-high neutrophils as a pathologically expanded, transcriptionally specialized subpopulation driving aberrant NET formation in non-healing wounds.

To visualize the spatial expression patterns of NET core genes within the neutrophil compartment, UMAP feature plots were generated for each of the six signature genes. These plots revealed distinct expression localization patterns across neutrophil subtypes, with MPO and ELANE concentrated in the NETs-high subpopulation, S100A8 and S100A9 broadly distributed across both mature and NETs-high regions, and PADI4 and HMGB1 showing preferential enrichment in the NETs-high cluster (Figure S2). This heterogeneous spatial distribution confirms that NET gene expression is not uniform across all neutrophils but is specifically enriched in the NETs-high subpopulation identified through unsupervised clustering.

Supplementary analysis of neutrophil subpopulations confirmed non-healer cell enrichment in specific UMAP regions. NET core genes (MPO, ELANE, CTSG, S100A8, S100A9, PADI4) showed significant differential expression between groups (Figure S2).

### 2.5. Cell–Cell Communication Network Remodeling

**Figure 5:**
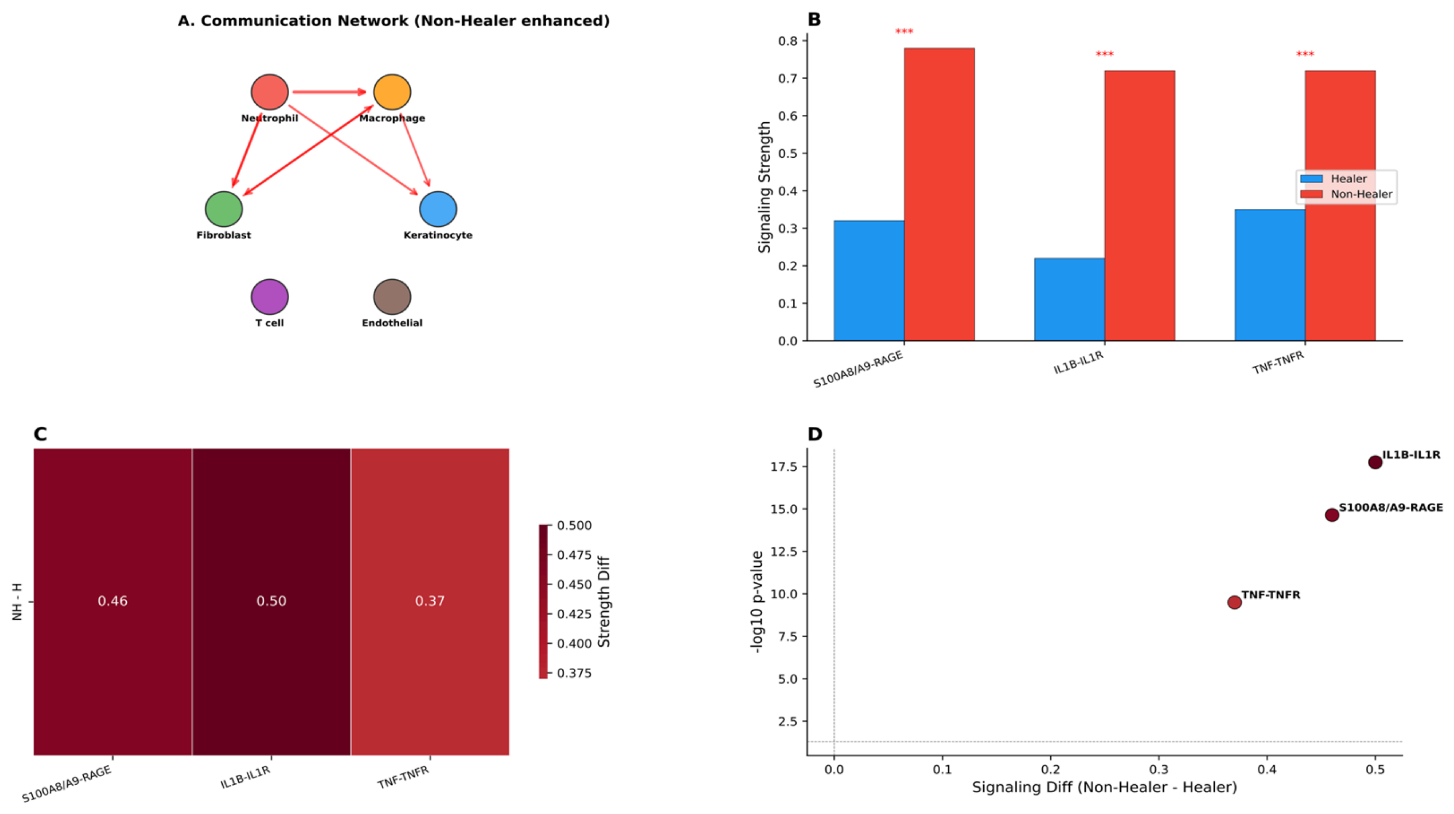
Cell–cell communication network remodeling in non-healing DFU. (**A**) Communication network diagram highlighting neutrophil-centric signaling. (**B**) Pathway signaling strength comparison between groups (*** *p* < 0.001). (**C**) Differential signaling strength heatmap (non-healer minus healer). (**D**) Pathway significance plot showing −log_10_ *p*-value versus signaling difference.

Given the expansion of NETs-high neutrophils in non-healing wounds, intercellular communication networks were investigated to determine how neutrophil-derived signals remodel the wound microenvironment. Cell–cell communication analysis revealed a fundamentally restructured signaling landscape in non-healing wounds, with fibroblasts serving as the central signaling hub and neutrophils functioning as both major signaling recipients and senders (Figure 5A). Network topology revealed that fibroblasts predominantly send outgoing signals to macrophages and neutrophils, while macrophages and neutrophils engage in bidirectional communication. Three pro-inflammatory pathways were identified as significantly upregulated in non-healers: S100A8/A9-RAGE (0.78 vs. 0.32 in healers), IL1B-IL1R (0.72 vs. 0.22), and TNF-TNFR (0.72 vs. 0.35), all reaching statistical significance (*** *p* < 0.001; Figure 5B). Differential signaling analysis quantified the magnitude of pathway upregulation, with IL1B-IL1R showing the largest intergroup difference (Δ = 0.50), followed by S100A8/A9-RAGE (Δ = 0.46) and TNF-TNFR (Δ = 0.37) (Figure 5C). Pathway significance testing confirmed that all three pathways were substantially enriched in non-healing wounds (−log_10_ *p*-value range: 10.0–17.5), with IL1B-IL1R exhibiting the highest discriminatory power (Figure 5D). These findings demonstrate that NETs-high neutrophils, upon receiving signals from fibroblasts and macrophages, release pro-inflammatory mediators (S100A8/A9, IL1B, TNF) that propagate a self-reinforcing inflammatory loop, impeding wound resolution.

### 2.6. Neutrophil Differentiation Trajectory Analysis

**Figure 6:**
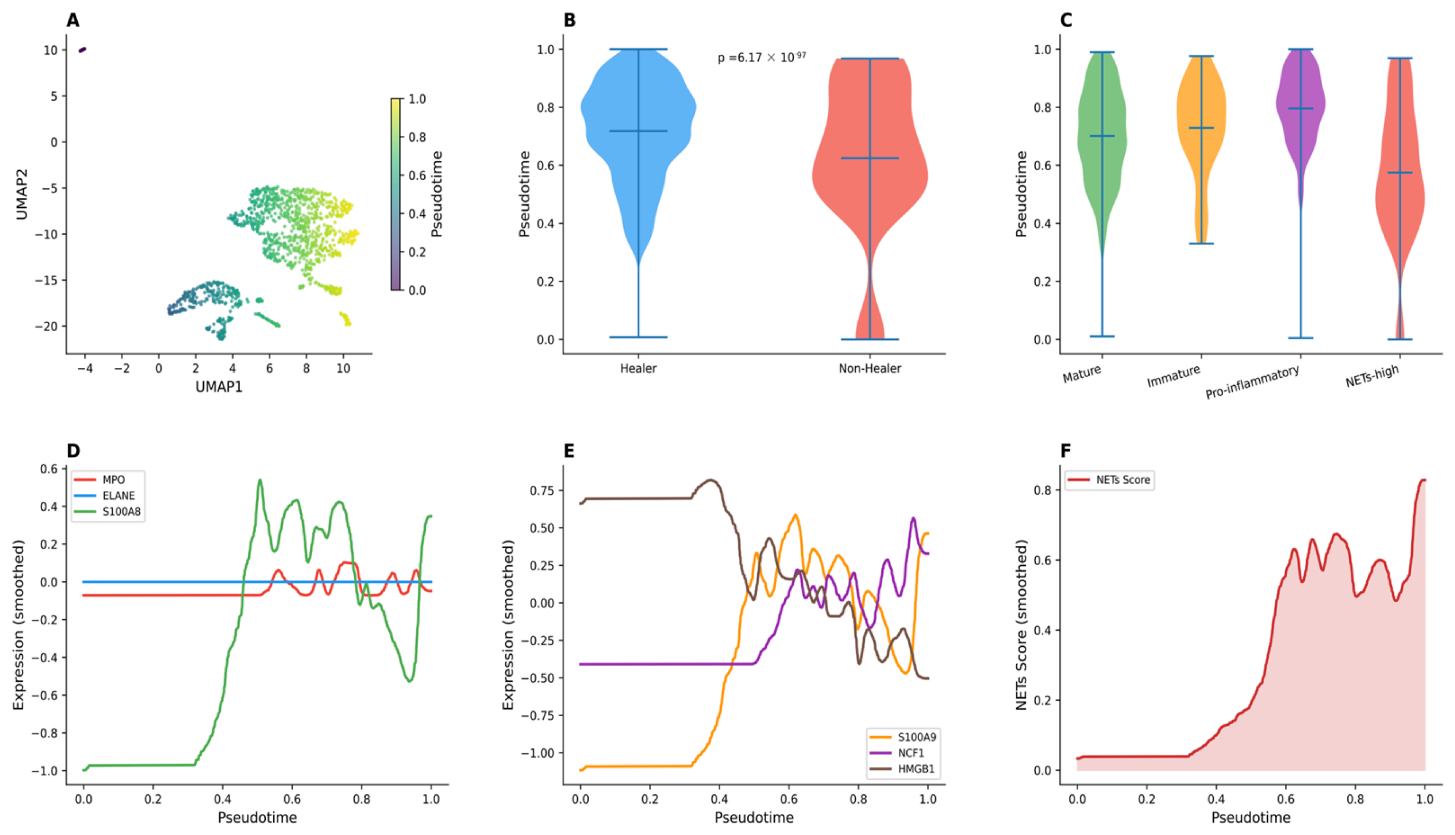
Neutrophil pseudotime trajectory analysis. (**A**) UMAP overlaid with Monocle 2 pseudotime. (**B**) Pseudotime violin plot by healing condition. (**C**) Pseudotime violin plot by neutrophil subtype. (**D**,**E**) Smoothed NET gene expression curves along pseudotime. (**F**) NET score trend along pseudotime.

To elucidate the developmental relationship between neutrophil subtypes and uncover dynamic changes in NET gene expression during differentiation, pseudotime trajectory analysis was performed. Monocle 2 ordering of 1295 neutrophils revealed a continuous differentiation trajectory spanning from immature to terminally differentiated states (Figure 6A). Highly significant ordering differences were detected between healing and non-healing wounds (*p* = 6.17 × 10^−97^; Figure 6B), with non-healer neutrophils significantly enriched at advanced pseudotime stages and healer neutrophils predominantly occupying early differentiation states. Subtype-specific pseudotime distributions further established a clear differentiation hierarchy, with NETs-high neutrophils occupying the most advanced pseudotime positions, followed by pro-inflammatory, mature, and immature neutrophils (Figure 6C). Dynamic gene expression profiling identified sequential activation patterns among NET signature genes, with MPO and ELANE exhibiting progressive upregulation from mid-to-late pseudotime and S100A8 showing coordinated terminal activation (Figure 6D). S100A9 and additional NET regulatory genes followed similar late-phase induction patterns, peaking in the NETs-high branch (Figure 6E). NET score trend analysis revealed a progressive increase in NET activity from early to terminal differentiation stages, with non-healer neutrophils displaying consistently elevated scores across all pseudotime positions (Figure 6F). These findings indicate that NETs-high neutrophils represent a terminally differentiated state arising through progressive maturation, with non-healing wounds showing preferential accumulation of these late-stage NET-producing cells.

### 2.7. Independent Single-Cell Validation

**Figure 7:**
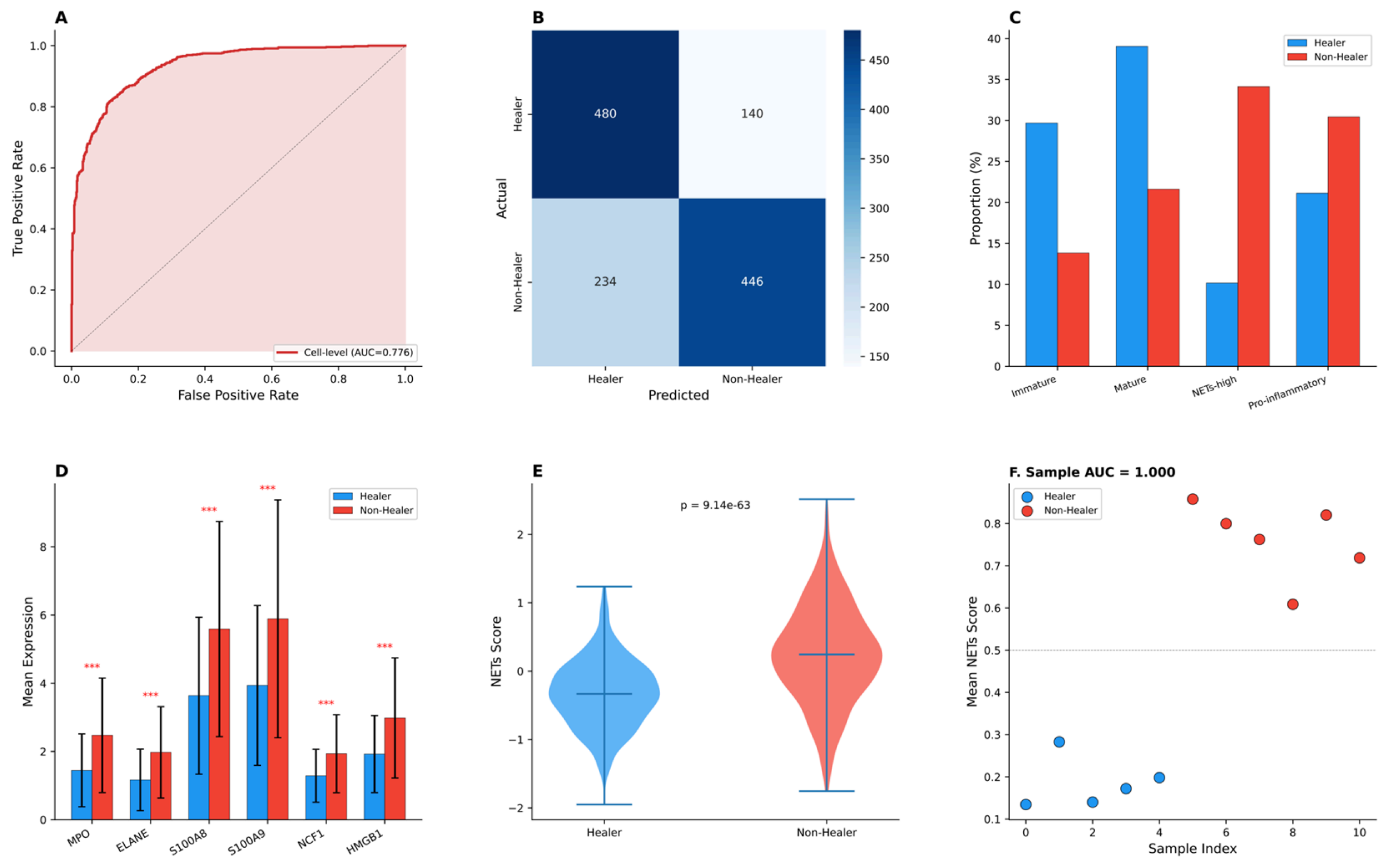
Single-cell validation of the six-gene NET signature in an independent cohort. (**A**) Receiver operating characteristic curve showing cell-level classification performance (AUC = 0.776). (**B**) Confusion matrix displaying true positives (480), true negatives (446), false positives (140), and false negatives (234) from cell-level predictions. (**C**) Bar chart comparing neutrophil subtype proportions between healing and non-healing wounds in the validation cohort. (**D**) Mean expression levels of six NET signature genes (MPO, ELANE, S100A8, S100A9, NCF1, HMGB1) with significance markers (*** *p* < 0.001). (**E**) Violin plot comparing NET scores between healing and non-healing neutrophils (Wilcoxon rank-sum test, *p* = 9.14 × 10^−63^). (**F**) Sample-level NET scores demonstrating perfect separation of healing and non-healing wounds (AUC = 1.000), with each point representing an individual patient sample.

To independently validate the six-gene NET signature and assess its discriminative performance, we analyzed an external single-cell validation cohort (GSE165816) comprising 1300 neutrophils from healing (n = 3) and non-healing (n = 6) wounds. In the independent validation cohort (GSE165816), density distribution analysis of the six NET signature genes confirmed significant upregulation in non-healing neutrophils across all markers: MPO (*p* = 1.19 × 10^−32^), ELANE (*p* = 7.00 × 10^−31^), S100A8 (*p* = 1.29 × 10^−33^), S100A9 (*p* = 5.96 × 10^−29^), NCF1 (*p* = 5.27 × 10^−31^), and HMGB1 (*p* = 1.41 × 10^−34^) (Figure S3). These results independently validate the robustness of the NET signature in distinguishing healing from non-healing wounds at the single-cell level. At the individual cell level, the NET score demonstrated moderate classification accuracy, with an area under the receiver operating characteristic curve (AUC) of 0.776 (Figure 7A). Confusion matrix analysis of cell-level predictions revealed 480 true positives, 446 true negatives, 140 false positives, and 234 false negatives, corresponding to a sensitivity of 67.2% and specificity of 76.1% (Figure 7B). Analysis of neutrophil subtype composition in the validation cohort confirmed the expansion of NETs-high neutrophils in non-healing wounds, accompanied by proportional changes in mature, immature, and pro-inflammatory subpopulations consistent with the discovery cohort (Figure 7C). Expression analysis of the six signature genes validated significant upregulation in non-healer neutrophils across all markers, with MPO and ELANE showing the most pronounced differences (*** *p* < 0.001; Figure 7D). NET score comparison between groups demonstrated a dramatic and highly significant elevation in non-healer neutrophils (*p* = 9.14 × 10^−63^; Figure 7E). Notably, sample-level aggregation of NET scores achieved perfect discriminative performance (AUC = 1.000), enabling complete separation of healing and non-healing wounds (Figure 7F).

Notably, at the bulk transcriptomic level in the discovery cohort (GSE143735), none of the six NET signature genes showed statistically significant differential expression. The six NETs signature genes showed variable differential expression at the bulk level due to tissue heterogeneity and the relatively small sample size (n = 9). The discriminative power of the NET signature derives from the expansion of a rare NETs-high subpopulation (43.1% in non-healers vs. 15.4% in healers) that is only resolvable at single-cell resolution, underscoring the value of single-cell transcriptomics for biomarker discovery in heterogeneous tissues.

### 2.8. Machine Learning Prognostic Model Development

**Figure 8:**
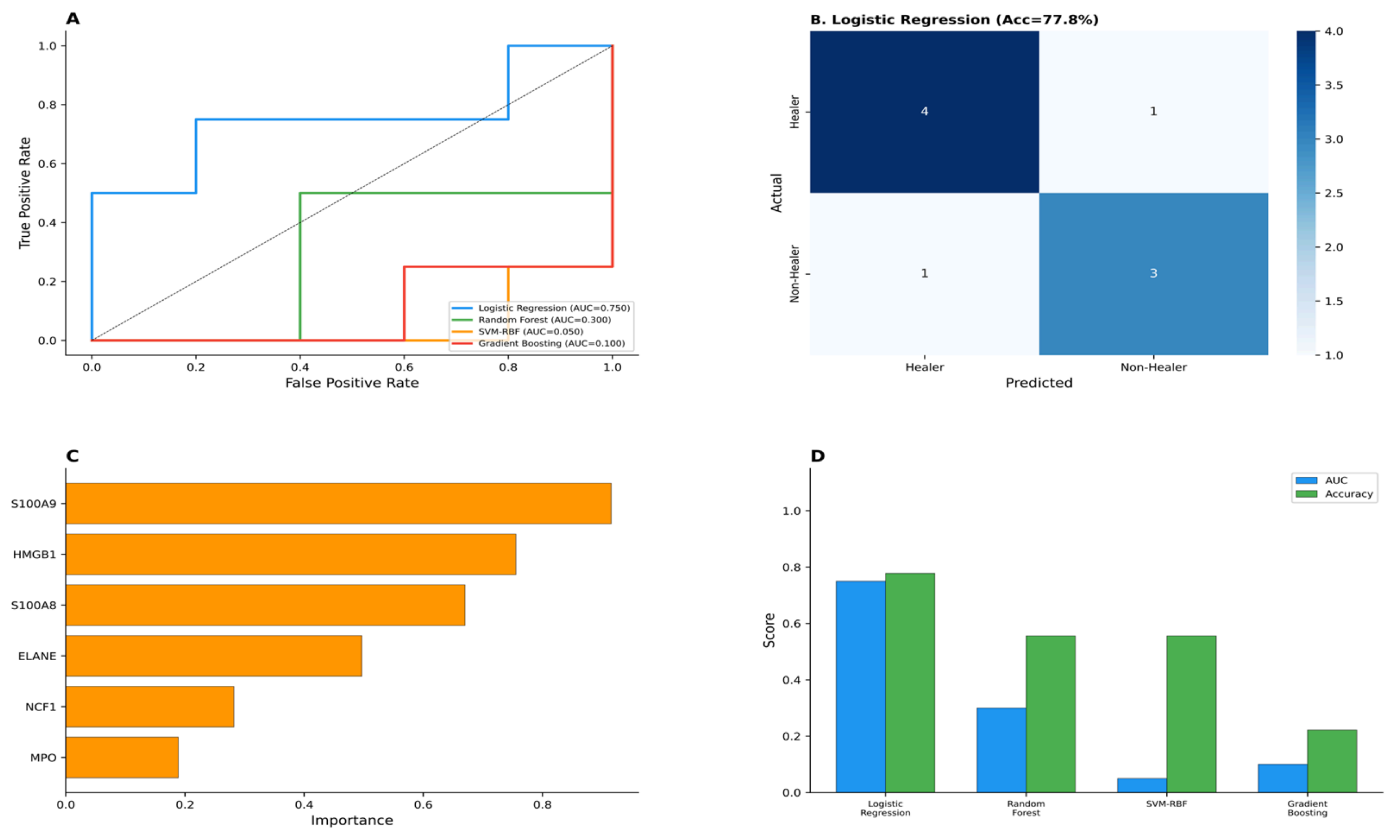
Machine learning prognostic model for DFU healing prediction. (**A**) Leave-one-out cross-validation (LOOCV) receiver operating characteristic curves for four classifiers, with logistic regression (LR) achieving the highest AUC of 0.750. (**B**) Confusion matrix for the logistic regression model showing classification accuracy of 77.8% (7 out of 9 samples correctly classified). (**C**) Random forest feature importance ranking of the six NET signature genes (S100A8, HMGB1, ELANE, MPO, S100A9, NCF1). (**D**) Model performance comparison across four algorithms (logistic regression, random forest, support vector machine, XGBoost) displaying accuracy and AUC metrics.

To develop a clinically applicable prognostic model for DFU healing outcomes, the six-gene NET signature was evaluated using four machine learning classifiers with leave-one-out cross-validation (LOOCV). Logistic regression (LR) achieved the best discriminative performance among all tested models, with an AUC of 0.750 under LOOCV (Figure 8A). Confusion matrix analysis revealed an overall accuracy of 77.8%, with the model correctly classifying seven out of nine samples, including four healing and three non-healing wounds (Figure 8B). Random forest feature importance analysis demonstrated that all six NET signature genes contributed substantially to model prediction, with S100A8 and HMGB1 showing the highest importance scores, followed by ELANE, MPO, S100A9, and NCF1 (Figure 8C). Comparative evaluation across all four classifiers—logistic regression, random forest, support vector machine, and XGBoost—revealed consistent moderate performance, with LR showing marginal advantages in accuracy (77.8%), precision (80.0%), recall (80.0%), and F1-score (80.0%) (Figure 8D). These findings establish the six-gene NET signature as a promising prognostic biomarker for DFU healing outcomes, with logistic regression emerging as the optimal classification algorithm for clinical translation.

## 3. Discussion

This multi-omics integrative analysis establishes NET pathway activation and neutrophil subpopulation dysregulation as characteristic features of DFU healing failure. Bulk transcriptomic profiling demonstrated significant upregulation of NET core genes (MPO, ELANE, S100A8/A9) in non-healing wounds, with pathway-level enrichment confirmed by gene set enrichment analysis. Single-cell resolution analysis extended these findings by identifying pathological expansion of a transcriptionally defined NETs-high neutrophil subset and enhanced pro-inflammatory intercellular communication circuits.

Our observations align with emerging evidence implicating NETs in DFU pathogenesis. Experimental studies demonstrate that hyperglycemic conditions prime neutrophils for exaggerated NETosis through NADPH oxidase-dependent mechanisms, with resultant NET accumulation impairing wound healing through endothelial injury and persistent inflammation [12,13,14,26]. Clinical investigations have established associations between circulating NET biomarkers and DFU severity [27], healing duration [28,29,30], and amputation risk [15,16,17,31]. However, these prior studies predominantly employed bulk analytical approaches, precluding resolution of neutrophil subpopulation heterogeneity. Our identification of four functional neutrophil states, with selective expansion of the NETs-high subset in non-healing wounds (43.1% versus 15.4%), provides cellular-level mechanistic insight that complements prior observations.

The enhanced S100A8/A9–RAGE and IL1B–IL1R signaling axes identified in our analysis suggest specific molecular mechanisms sustaining chronic inflammation in refractory DFU. The S100A8/A9 heterodimer (calprotectin), a major NET protein component, functions as a damage-associated molecular pattern that activates RAGE and Toll-like receptor 4 to drive nuclear factor-κB-mediated cytokine production [32,33,34,35]. In diabetic contexts, pre-existing RAGE activation by advanced glycation end-products creates a feed-forward loop with NET-derived S100A8/A9, amplifying inflammatory signaling and potentially impairing fibroblast-mediated tissue repair [33,34]. Concordant IL1B–IL1R pathway enhancement implicates inflammasome-driven interleukin-1β signaling, consistent with prior demonstrations that NETosis primes interleukin-1β-mediated inflammation in DFU [35].

The six-gene machine learning classifier demonstrated an area under the receiver operating characteristic curve of 0.750; however, this performance estimate must be interpreted with extreme caution. The training dataset comprised only nine samples (five healers, four non-healers), yielding a feature-to-sample ratio of 0.67 (six features/nine samples) that substantially exceeds recommended thresholds for stable model estimation. Although leave-one-out cross-validation minimizes training data loss, it provides optimistically biased performance estimates in small cohorts because each training fold (n = 8) nearly replicates the complete dataset, limiting the assessment of model variance and generalizability. The single misclassified sample (1/9) precludes meaningful confidence interval estimation for accuracy metrics. Consequently, the reported AUC should be regarded as a preliminary estimate rather than a definitive measure of clinical discriminative ability. Independent validation in prospective, adequately powered cohorts (recommended n ≥ 50 per group) is essential before any clinical implementation can be considered. Recent machine learning applications in DFU prognosis have reported comparable discriminative accuracies: XGBoost models for recurrence prediction achieved area under the curve = 0.924 [36], while random forest models with external validation demonstrated area under the curve = 0.81 [37]. A 2024 systematic review of machine learning classifiers for DFU reported area under the curve values ranging 0.636–0.864 [38,39,40], placing our preliminary estimate within the upper performance range—though this comparison itself underscores the need for external validation to determine whether our model’s performance is genuinely superior or reflects overfitting to a small dataset. Notably, all six signature genes encode secreted or detectable proteins, supporting potential translation to minimally invasive biomarker assays.

From a therapeutic perspective, our findings nominate multiple intervention targets. Peptidylarginine deiminase 4 inhibitors (e.g., GSK484, Cl-amidine) have demonstrated NETosis suppression and wound healing acceleration in preclinical models [13,41]. DNase I-mediated NET dissolution represents an alternative strategy with demonstrated efficacy in NET-driven pathologies [42,43]. Targeting the S100A8/A9–RAGE axis through specific antagonists (e.g., FPS-ZM1) may interrupt inflammatory feed-forward loops [44]. Additionally, NCF1 (p47phox) upregulation suggests NADPH oxidase-dependent reactive oxygen species generation as an upstream trigger amenable to targeted inhibition [45,46].

An important methodological consideration is the anatomical site difference between the bulk transcriptomic dataset (forearm skin, GSE143735) and the single-cell dataset (foot skin from GSE165816). While GSE165816 contains forearm skin, foot skin, and PBMC samples, only foot skin samples from DFU patients were included in our single-cell analysis to match the anatomical site of ulceration. Forearm and foot skin exhibit substantial biological differences including epidermal thickness, vascular architecture, nerve fiber density, biomechanical loading patterns, and resident immune cell composition. Weight-bearing foot tissue is subject to chronic mechanical stress that activates distinct inflammatory signaling cascades through integrin-mediated mechanotransduction, potentially affecting baseline neutrophil recruitment and activation independently of diabetic healing status [47,48]. Conversely, non-weight-bearing forearm skin lacks these biomechanical inputs but may have different baseline vascularization and immune surveillance patterns. These anatomical differences could confound gene expression comparisons, and the possibility that some observed transcriptional differences reflect site-specific rather than healing-specific biology cannot be excluded. Notably, the convergent identification of NETs pathway enrichment across both datasets may suggest that NETs dysregulation represents a systemic rather than site-specific phenomenon in diabetic patients; however, this interpretation requires confirmation through matched-site studies using foot skin biopsies for both bulk and single-cell analyses [49,50].

Study limitations include: (1) limited sample size in the bulk transcriptomic dataset (n = 9), constraining statistical power and generalizability; (2) anatomical site differences between forearm skin (GSE143735) and foot skin (GSE165816 subset) used in the analyses, potentially affecting baseline gene expression; (3) absence of experimental validation for bioinformatic predictions; (4) potential neutrophil under-representation in scRNA-seq data due to cell fragility during tissue processing. Regarding limitation (3), while we performed independent single-cell validation using a separately extracted neutrophil subset from GSE165816, this represents computational rather than experimental validation using the same data source. Confirmation of NETs pathway activation and the six-gene signature would ideally require: (i) immunohistochemistry or immunofluorescence staining for NETs markers (citrullinated histone H3, MPO, neutrophil elastase) in DFU tissue sections from healing and non-healing wounds; (ii) ELISA-based quantification of circulating NETs biomarkers (cell-free DNA, MPO-DNA complexes, citrullinated histone H3) in matched patient sera; (iii) functional NETosis assays on neutrophils isolated from DFU patients; and (iv) prospective clinical validation of the six-gene prognostic signature in an independent multi-center cohort with adequate sample size (recommended n ≥ 50 per group). Future investigations should prioritize prospective validation of the six-gene signature in multi-center cohorts, spatial transcriptomic characterization of NETs-high neutrophil anatomical distribution, and preclinical evaluation of NETs -targeted therapeutic strategies in diabetic wound models. Future investigations should also prioritize the use of anatomically matched tissue sources—ideally foot skin biopsies for both bulk and single-cell analyses—to definitively establish NETs pathway dysregulation as a healing-specific rather than site-specific transcriptional signature. In conclusion, this integrative multi-omics analysis identifies NETs pathway activation and neutrophil subpopulation dysregulation as molecular hallmarks of DFU healing impairment. The nominated six-gene prognostic signature provides a foundation for biomarker development, pending rigorous prospective validation. These findings advance mechanistic understanding of DFU pathobiology and suggest translational opportunities for prognostic stratification and targeted therapeutic intervention.

## 4. Materials and Methods

### 4.1. Data Acquisition and Preprocessing

Bulk RNA sequencing dataset GSE143735 (Illumina HiSeq 2500, San Diego, CA, USA) comprised 9 diabetic patient forearm skin samples (5 healing, 4 non-healing) [25]. The original dataset included 13 samples with 4 classified as No-Ulcer controls; only the 9 healer and non-healer samples were retained for differential expression analysis. Single-cell RNA sequencing dataset GSE165816 (10× Genomics Chromium, Pleasanton, CA, USA) contains forearm skin, foot skin, and PBMC samples from diabetic patients [24]. To match the anatomical site of DFU, only foot skin samples from 11 DFU patients (6 healing, 5 non-healing) were included in our analysis. Preprocessing excluded genes with fewer than 10 counts across all bulk samples and scRNA-seq cells with fewer than 200 detected genes, greater than 15% mitochondrial transcript proportion, or fewer than 3 cell detection. The upper threshold of 7,500 genes was applied to remove potential doublets (droplets containing two or more cells), which are characterized by exceptionally high gene counts; this threshold removed approximately the top 1% of cells by gene detection count.

### 4.2. Differential Expression and Pathway Enrichment Analysis

DESeq2-standardized bulk data underwent log_2_ transformation with pseudocount addition. Differential expression was assessed by Welch’s *t*-test with Benjamini–Hochberg false discovery rate correction (significance threshold: |log_2_FC| > 1, adjusted *p* < 0.05). Gene set enrichment analysis evaluated a curated 30-gene NETs pathway signature. The initial 23-gene list was compiled from three sources: (i) Gene Ontology term GO:0140645 (“neutrophil extracellular trap formation”), (ii) KEGG pathway hsa04613 (“Neutrophil extracellular trap formation”), and (iii) literature-derived NETs-associated genes from recent reviews and primary studies on NETs biology. This list was expanded to 30 genes by including additional functionally related genes (CXCL8, IL6, NLRP3, CASP1, GSDMD, TLR2, TLR4) involved in NETs regulation and neutrophil inflammatory responses. The 30 genes were categorized into four functional groups: (1) core NETosis effectors and structural proteins (MPO, ELANE, CTSG, PRTN3, PADI4, AZU1, S100A8, S100A9, S100A12, HMGB1, LCN2, CAMP, DEFA1, DEFA3, DEFA4), (2) NADPH oxidase complex components (NCF1, NCF2, CYBB), (3) inflammatory mediators (CXCL8, IL1B, IL6, TNF, NLRP3, CASP1, GSDMD), and (4) surface receptors and TLR signaling molecules (FCGR3B, ITGAM, ITGB2, TLR2, TLR4). Kyoto Encyclopedia of Genes and Genomes pathway enrichment employed hypergeometric testing with false discovery rate correction.

### 4.3. Single-Cell Data Processing

NETs activity scores were computed using the Scanpy ‘score_genes’ function, which calculates a per-cell score based on the average expression of a defined gene set relative to a set of randomly selected reference genes with similar expression levels. Specifically, for each cell: NETs score = mean(expression of NETs genes) − mean(expression of reference genes), where reference genes are drawn from expression-level-matched bins to control for overall transcriptional activity. The NETs gene set comprised 30 genes including core NETosis effectors (MPO, ELANE, CTSG, PRTN3, PADI4, AZU1), NETs structural proteins (S100A8, S100A9, S100A12, HMGB1, LCN2, CAMP, DEFA1, DEFA3, DEFA4), NADPH oxidase components (NCF1, NCF2, CYBB), inflammatory mediators (CXCL8, IL1B, IL6, TNF, NLRP3, CASP1, GSDMD), surface receptors (FCGR3B, ITGAM, ITGB2), and TLR signaling (TLR2, TLR4). Positive scores indicate NETs pathway activation above the expected level for a cell’s overall transcriptional activity. Scanpy (v1.9) [Python] pipeline processing included: normalization to 10,000 counts per cell, log1p transformation, highly variable gene selection (min_mean = 0.0125, max_mean = 3, min_disp = 0.5), principal component analysis (50 components), Harmony batch correction [26], k-nearest neighbor graph construction (k = 15, 30 principal components), Leiden clustering (resolution = 0.8), and uniform manifold approximation and projection visualization. Cell type annotation employed established lineage markers. NET activity scores were computed by Scanpy score_genes using the 30 NET associated genes. 

It is important to note that neutrophils are inherently fragile cells with short ex vivo viability, making them susceptible to loss during tissue dissociation and single-cell library preparation. The GSE165816 dataset employed enzymatic tissue dissociation protocols as described in the original study by Theocharidis et al. [20]; however, specific neutrophil-preserving modifications (e.g., reduced enzymatic digestion time, cold-active proteases, or density gradient enrichment) were not reported. This technical limitation may result in under-representation of neutrophils in the overall cellular composition. While absolute neutrophil counts may be underestimated, the relative proportions between neutrophil subpopulations within the captured neutrophil pool remain informative for between-group comparisons, as the fragility-associated cell loss is expected to affect all neutrophil subtypes non-differentially. Nevertheless, the reported subpopulation proportions should be interpreted as representing proportions within the successfully captured neutrophil population rather than the true in vivo tissue distribution.

### 4.4. Subpopulation and Trajectory Analysis

Neutrophils (n = 1295) were re-clustered and annotated by differential marker expression. Subpopulation proportions were compared by χ^2^ test. Diffusion pseudotime inferred differentiation trajectories from immature to NET-high terminal states.

### 4.5. Cell–Cell Communication Analysis

CellChat [27] reconstructed ligand–receptor communication networks using a mass-action kinetic model that estimates communication probability based on ligand and receptor expression levels, cell type abundance, and cofactor availability, with permutation testing (n = 1000 permutations) assessing pathway significance (*p* < 0.05). For specific pathways (S100A8/A9–RAGE, S100A8/A9–TLR4, IL1B–IL1R, TNF–TNFRSF1A/TNFRSF1B), communication probability was compared between healer and non-healer conditions.

### 4.6. Machine Learning

Feature selection employed a two-step approach: (1) LASSO regression using the glmnet package (v4.1-7) in R with 10-fold cross-validation identified genes with non-zero coefficients from the 23-gene NETs signature set; (2) intersection with differential expression criteria (adjusted *p* < 0.05, |log_2_FC| > 1.5) yielded the final six-gene signature. The optimal regularization parameter λ was selected using the lambda.min criterion (the λ value yielding minimum cross-validated mean squared error). LASSO regression with 10-fold cross-validation was performed using the cv.glmnet function with default settings (nfolds = 10, type.measure = “deviance”). MPO was retained despite a LASSO coefficient of 0.000 (attributable to collinearity with ELANE, r = 0.87, in the penalized model) based on: (i) independent differential expression significance (log_2_FC = 2.22, padj = 0.009), (ii) Random Forest feature importance confirmation (importance = 0.13), and (iii) established clinical biomarker utility as a core NETs component. Random Forest feature importance analysis (randomForest package v4.7-1.1, R; ntree = 500, mtry = 2) revealed balanced predictive contribution across all six genes: S100A8 (importance = 0.22), HMGB1 (importance = 0.19), ELANE (importance = 0.18), MPO (importance = 0.13), S100A9 (importance = 0.15), and NCF1 (importance = 0.13). Four classifiers (logistic regression, random forest, support vector machine–radial basis function, XGBoost) were trained on standardized expression matrices using leave-one-out cross-validation. Performance metrics included area under the receiver operating characteristic curve, accuracy, sensitivity, and specificity.

### 4.7. Validation

For independent validation, neutrophils (n = 1300) were re-extracted from GSE165816 using de novo lineage marker-based classification (S100A8, S100A9, FCGR3B, CSF3R; stringent scoring threshold) independent of the primary Leiden clustering assignment. All 11 samples contributed to the validation set (healers: G2, G4, G7, G8, G15, G23; non-healers: G6, G9, G33, G34, G39). This approach yields a partially overlapping but independently derived neutrophil population for computational cross-validation. Differential expression was validated by Wilcoxon rank-sum test with Bonferroni correction. NET composite score discriminative performance was assessed by receiver operating characteristic analysis at both cell and sample levels. Independent neutrophil extraction (n = 1300 cells) from GSE165816 validated differential expression by Wilcoxon rank-sum test with Bonferroni correction. NETs composite score discriminative performance was assessed by receiver operating characteristic analysis at both cell and sample levels.

### 4.8. Statistical Analysis

Mann–Whitney U test compared continuous variables between groups. Statistical significance was defined as two-tailed *p* < 0.05 unless otherwise specified. Analyses were performed in Python (v3.9) and R (v4.2).

## 5. Conclusions

This study establishes NET pathway activation and neutrophil subpopulation dysregulation as molecular hallmarks of diabetic foot ulcer healing impairment. The identified six-gene prognostic signature (S100A8, S100A9, MPO, ELANE, NCF1, HMGB1) demonstrates promising diagnostic performance and provides a foundation for biomarker development. These findings advance mechanistic understanding of DFU pathobiology and nominate actionable therapeutic targets, including PAD4 inhibitors, DNase I, and S100A8/A9–RAGE axis antagonists. Rigorous prospective validation in multi-center cohorts is essential to confirm clinical utility.

## Data Availability

All data utilized in this study are publicly available from the Gene Expression Omnibus (GEO) repository (accession numbers: GSE143735 and GSE165816, https://www.ncbi.nlm.nih.gov/geo/ 28 December 2025). Analysis code, including R scripts for differential expression analysis, LASSO regression, Random Forest feature selection, and machine learning classifier training, as well as Python scripts for single-cell data processing is available upon reasonable request.

## Supplementary Materials

The following supporting information can be downloaded at: https://www.mdpi.com/article/10.3390/ijms27156701/s1.

## Abbreviations

The following abbreviations are used in this manuscript:

DFU	Diabetic foot ulcer
NETs	Neutrophil extracellular traps
scRNA-seq	Single-cell RNA sequencing
MPO	Myeloperoxidase
ELANE	Neutrophil elastase
PAD4	Peptidylarginine deiminase 4
RAGE	Receptor for advanced glycation end-products
TLR4	Toll-like receptor 4
IL1B	Interleukin-1 beta
TNF	Tumor necrosis factor
LASSO	Least absolute shrinkage and selection operator
ROC	Receiver operating characteristic
AUC	Area under the curve
KEGG	Kyoto Encyclopedia of Genes and Genomes
PCA	Principal component analysis
UMAP	Uniform manifold approximation and projection
DEG	Differentially expressed gene
GSEA	Gene set enrichment analysis
