# Supplementary material for: Multi-Omics Profiling Reveals Neutrophil Extracellular Trap Dysregulation in Diabetic Foot Ulcer Healing Impairment
Source: Int J Mol Sci. 2026 Jul 27;27(15):6701. doi: 10.3390/ijms27156701 (PMC13467576; doi:10.3390/ijms27156701)
Supplement: Supplementary file 1 [file ijms-27-06701-s001.zip › ijms-4201368-supplementary.pdf]

Supplementary Materials:

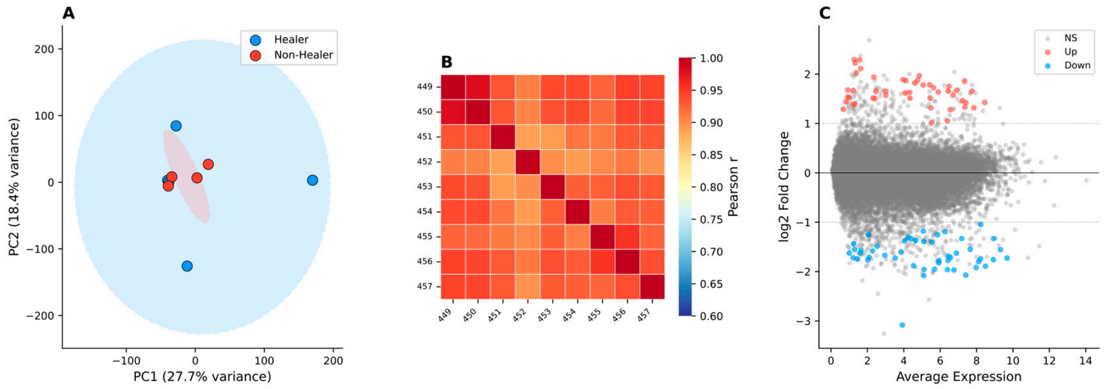

•Figure S1:Data Quality Control and Global Transcriptomic Signatures (A) PCA analysis showing sample distribution between healer (blue) and non-healer (red) groups with 95% confidence ellipses (PC1 = 27.7%, PC2 = 18.4%); (B) Sample Pearson correlation heatmap; (C) MA plot showing differential gene distribution (red: upregulated, blue: downregulated).

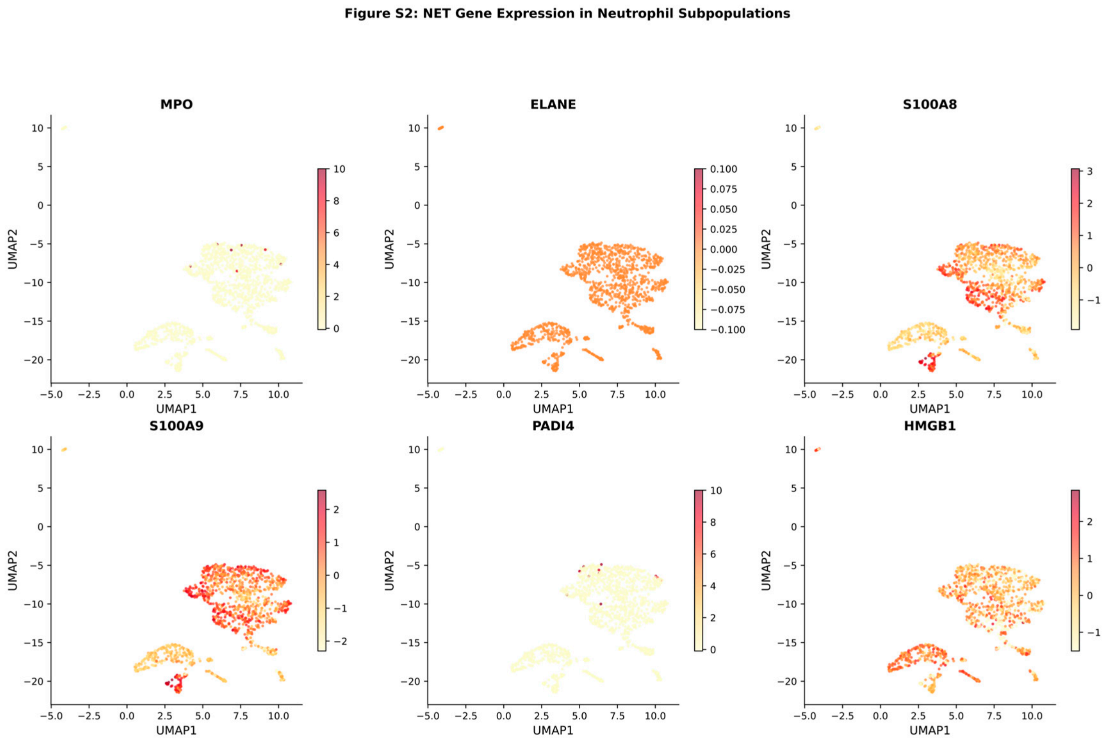

• Figure S2: UMAP feature plots showing expression distribution of six NETs signature genes across neutrophil subpopulations in diabetic foot ulcer: MPO, ELANE, S100A8, S100A9, PADI4, and MGB1. Each panel displays 1,295 neutrophils projected onto UMAP coordinates, with color intensity indicating normalized gene expression level (yellow: low, red: high). MPO, ELANE, and PADI4 are concentrated in the NETs-high subpopulation (upper-right cluster), while S100A8 and S100A9 show broader expression across activated neutrophil states.

Figure S3: Key Gene Expression in Validation Cohort

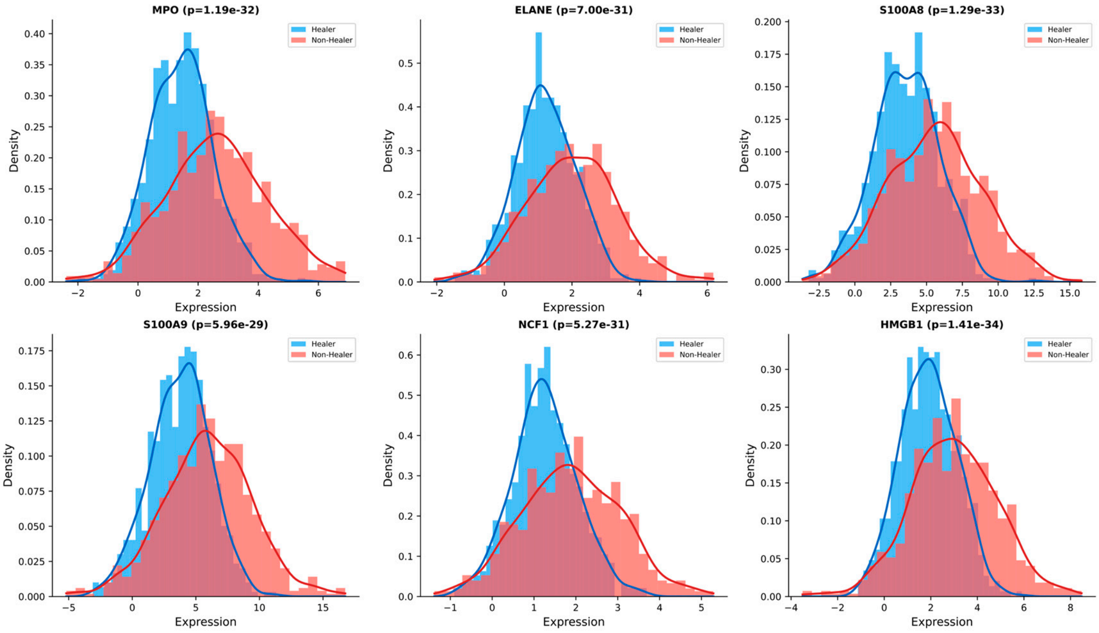

• Figure S3: Histogram and kernel density estimation plots showing expression distributions of MPO, ELANE, S100A8, S100A9, NCF1, and HMGB1 in healing (blue, n = 620) and non-healing (red, n = 680) neutrophils. p-values from Wilcoxon rank-sum test with Bonferroni correction

Figure S4: 6-Gene Signature Expression in GSE143735

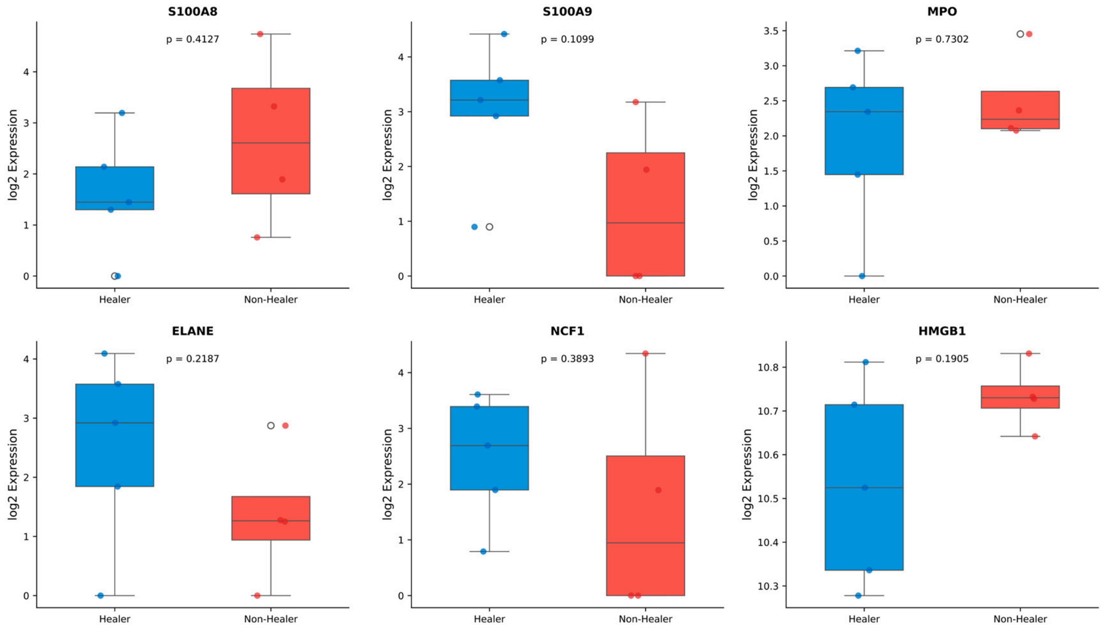

• Figure S4: Expression distribution analysis of six NETs core genes in validation cohort. Box-strip plots showing expression distributions of S100A8, S100A9, MPO, ELANE, NCF1, and HMGB1 in healer (blue, n = 5) and non-healer (red, n = 4) samples. p-values from Mann–Whitney U test.
